# Effects of Endophytic Fungi and Arbuscular Mycorrhizal Fungi on Microbial Community Function and Metabolic Pathways in the Rhizosphere Soil of *Festuca rubra*

**DOI:** 10.3390/microorganisms13122735

**Published:** 2025-11-30

**Authors:** Zhengming Luo, Yanying Zhou, Xuerong Wang, Lei He, Tong Jia

**Affiliations:** 1 Department of Geography, Xinzhou Normal University, Xinzhou 034000, China; 2 Soil Health Laboratory of Shanxi Province, Shanxi Agricultural University, Taiyuan 030031, China; 3 Shanxi Key Laboratory for Ecological Restoration of Loess Plateau, Institute of Loess Plateau, Shanxi University, Taiyuan 030006, China; 4 Shanxi Forestry and Grassland General Engineering Station, Taiyuan 030021, China

**Keywords:** endophytic fungi, arbuscular mycorrhizal fungi, *Festuca rubra*, microbial community

## Abstract

Numerous studies have shown that there are many uncertainties associated with the interactions of nitrogen with plants and microorganisms. In particular, the effects of symbioses between plants and various microorganisms on soil microbial community function remain unclear. Metagenomic sequencing was used to explore the changes in microbial community composition, function and metabolic pathways in rhizosphere soil and the associated influencing factors under different nitrogen levels caused by arbuscular mycorrhizal fungi (AMF) inoculation of *F. rubra* infected with endophytic fungi and nonendophytic fungi. Plant nutrient allocation (aboveground/belowground), soil pH, and enzymatic activities significantly modulated the functional profiles of the bacterial, fungal, and archaeal communities within these rhizospheres. Soil *β*-glucosidase activity had the greatest effect on the cluster of orthologous groups of proteins (COG) function of the rhizosphere soil bacterial community, and soil L-leucine aminopeptidase had the greatest effect on the COG function of the rhizosphere soil fungal and archaeal communities. The contributions of AMF colonization to the kyoto encyclopedia of genes and genomes (KEGG) functions of bacteria and archaea in the rhizosphere soil were greater than those of *F. rubra* infection with endophytic fungi, and AMF colonization improved the metabolic pathways, secondary metabolite biosynthesis, microbial metabolism, amino acid biosynthesis and carbon metabolism of bacterial and archaeal communities in the rhizosphere soil of *F. rubra*. The effects of endophytic fungi and AMFs on the function and metabolic pathways of symbiotic rhizosphere soil microbial communities were heterogeneous. This study revealed that considering both biotic and abiotic factors is essential for predicting the maintenance of soil ecosystem function by plant–fungal symbionts.

## 1. Introduction

In recent years, the accelerated utilization of fossil fuels and synthetic nitrogen fertilizers has resulted in elevated nitrogen loading within terrestrial and aquatic ecosystems. Simultaneously, climate change has progressively increased the rate of atmospheric nitrogen deposition on a global scale, with clear implications for ecosystem biogeochemical cycles [1]. Nitrogen plays an important role in plant physiological metabolism as an essential nutrient for plant growth, not only affecting plant growth and development and community composition but also critically affecting the characteristics of rhizosphere soil microbial communities [2,3], which are highly important for maintaining plant and soil biodiversity and ecosystem stability.

Endophytic fungi, which reside within plant tissues for most or all of their life cycle without causing significant disease symptoms in the host [4], play a notable role in shaping soil microbial communities. Research indicated that these fungi could modify soil microbial characteristics by enhancing the availability of nutrients such as soil organic carbon, total nitrogen [5], phenolic compounds, and organic acids [6]. Additionally, endophytic fungal infection improved soil pH, boost bacterial abundance, reduced fungal abundance, altered fungal community composition, and increased overall microbial diversity [5]. But other studies present a contrasting perspective, noting that while endophyte infection stimulates microbial activity, it did not necessarily lead to significant shifts in microbial community structure [7].

Arbuscular mycorrhizal fungi (AMFs) form symbiotic relationships with approximately 90% of terrestrial plants, including flowering plants, bryophytes, and ferns [8]. AMFs improve nutrient utilization and uptake by roots by forming a broad network of bacterial filaments in trophic zones outside the rhizosphere [9] and increasing plant resistance to drought, salt and disease [10]. Simultaneously, AMFs can modulate the soil microbial community composition and increase plant tolerance to abiotic stress Via the regulation of root physiological processes, including the enhancement of secondary metabolite biosynthesis and the upregulation of antioxidant enzyme activities [11]. In addition, the abundances and diversities of soil bacteria and archaea decrease after AMF inoculation of different plants [12], whereas soil eukaryotes respond more strongly to plant-mediated effects of AMFs than bacteria do. AMFs are key drivers of soil function [13] and can improve microbial versatility by regulating rhizosphere fungal diversity and soil properties [14].

In nature, many plants are ubiquitously cocolonized by both endophytic fungi and AMFs [8,10]. AMFs directly and indirectly modulate soil microbial community structures primarily through their symbiotic associations with plants, altering microbial diversity and community composition [13]. Although the effects of endophytic fungi and AMFs on soil microbial community structure have been studied extensively [5,6], the relative contributions of endophytic fungi and AMFs to the functions of symbiotic rhizosphere soil microbial communities remain unclear [11,12]. *Festuca rubra* is a perennial herbaceous plant with slender leaves and well-developed roots that is characterized by strong stress resistance, a high growth rate, and strong adaptability [5,7]. With respect to slope ecological restoration in Southwest China, *F. rubra* is mostly used as a pioneer species [15]. The endophytic fungus that infects *F. rubra* is *Epichloë festucae*, which has a high infection rate; members of this genus can infect a variety of cold-season grasses. In a previous investigation, the colonization rate of *F. rubra* with this endophytic fungus was 100%, and the roots were successfully colonized by AMFs, as evidenced by the presence of arbuscules, vesicles, and intraradical hyphal networks, which are key morphological indicators of established mycorrhizal symbiosis [16]. In recent years, with increasing research on *F. rubra* worldwide, the effects of endophytic fungi and AMFs on the characteristics of the rhizosphere microbial community of *F. rubra* have received considerable attention.

For this investigation, a controlled pot experiment incorporating biotic and abiotic parameters implemented with a completely randomized block design was performed, and three experimental variables were manipulated: nitrogen level, endophytic fungal infection, and AMF colonization. This research focused on elucidating the mechanisms by which dual inoculation with endophytic fungi and AMFs influences the structural and functional assembly of *F. rubra* rhizosphere microbial communities under various nitrogen regimens. Specifically, our hypothesis was that there would be a trade-off between endophytic fungi and AMFs in terms of the effects on different functions of rhizosphere soil microbial communities under dual colonization. This study serves as a theoretical basis for understanding the synergistic mechanisms of plants and microorganisms in the context of global climate change and provides a scientific basis for clarifying the relative contributions of different microbial groups to the functions of the symbiotic rhizosphere soil microbial community.

## 2. Materials and Methods

### 2.1. Experimental Design

The endophytic fungus that infects *F. rubra* is *Epichloë festucae* [15], which has a colonization rate of more than 90%. The endophytic fungal colonization rate of *F. rubra* seeds was determined by microscopic examination before the experiment, and the seed surfaces were sterilized before planting by soaking in 10% H_2_O_2_ solution for 10 min and then rinsing with sterile water. The seeds were dried at 60 °C for one month to obtain seeds that were not infected with endophytic fungi.

All experiments in this study were conducted at ambient temperature. The plant growth substrate was prepared by mixing river sand and zeolite at a 2:1 ratio after passing through a 2 mm sieve. The mixture was sterilized in an autoclave at 121 °C and 0.11 MPa for 2 h to create a sterile environment and eliminate the influence of other microorganisms. Proliferation of the *Rhizophagus irregularis* (RI) was carried out by planting the host plant *Sorghum bicolor*. For AMF treatments, a 100 g dose of AMF inoculum was applied Via layered incorporation, and 100 g of high-temperature-sterilized RI microbial agent was added to the control treatment mixture to ensure that only the target species (RI) and no other microorganisms were present at the beginning of the experiment.

A completely randomized block design was adopted with three variables: nitrogen, AMF, and endophytic fungi. Specifically, the treatments included RI colonization and no RI colonization (NM), endophytic fungi infected (EI) and endophytic fungi free (EF), and three nitrogen supplementation levels, namely, high nitrogen (HN, 3 g/L), low nitrogen (LN, 0.3 g/L), and no nitrogen (N0, 0 g/L), with three replicates for each treatment, resulting in a total of 36 potted plants. Before the experimental treatment, 15 *F. rubra* plants were grown in each pot. Hoagland nutrient solution was periodically applied to the soil to sustain healthy plant growth. Each pot received 500 mL of a modified Hoagland nutrient solution every two weeks to ensure a consistent nutritional baseline across all treatments. The Hogland nutrient solution contained 5.0 mM CaCl_2_, 5.0 mM KCl, 2.5 mM MgSO_4_·7H_2_O, 2.0 mM KH_2_PO_4_, 29 μM Na_2_-EDTA, 20 mM FeSO_4_·7H_2_O and trace elements 45 mM H_3_BO_3_, 6.6 mM MnSO_4_, 0.8 mM ZnSO_4_·7H_2_O, 0.6 mM H_2_MoO_4_, 0.4 mM CuSO_4_·5H_2_O. After a 90-day cultivation period, the mycorrhizal colonization rate, plant growth, and physiological parameters were measured.

### 2.2. Nutritional Characteristics of F. rubra and Determination of Rhizosphere Soil Enzyme Activity

The contents of total carbon (STC), total nitrogen (STN) and total sulfur (STS) in the aboveground part of *F. rubra* and the contents of total carbon (STC), total nitrogen (RTN) and total sulfur (RTS) in the belowground part of *F. rubra* were determined Via an elemental analyzer (vario MACRO cube, Langenselbold, Germany). N-acetyl-β-D-glucosidase (S-NAG), β-glucosidase (β-GC), L-leucine aminopeptidase (L-LAP), neutral phosphatase (NP), polyphenol oxidase (PPO), and peroxidase (POD) activities were determined Via an enzyme-linked immunoassay (ELISA).

### 2.3. DNA Extraction, Library Construction, and Metagenomic Sequencing

Total genomic DNA was extracted from the soil samples using the E.Z.N.A. Soil DNA Kit (Omega Biotek, Norcross, GA, USA) according to the manufacturer’s instructions. The concentration and purity of the extracted DNA were determined with a TBS-380 and NanoDrop2000, respectively. The quality of the extracted DNA was checked on a 1% agarose gel. The extracted DNA was fragmented to an average size of approximately 400 bp Via a Covaris M220 (Gene Company Limited, Shenzheng, China) for paired-end library construction. A paired-end library was constructed using NEXTFLEX^®^ Rapid DNA-Seq (Bioo Scientific, Austin, TX, USA). Adaptors containing the full complement of the sequencing primer hybridization sites were ligated to the blunt ends of the fragments. Paired-end sequencing was performed on an Illumina NovaSeq 6000 (Illumina Inc., San Diego, CA, USA) at Majorbio Bio-Pharm Technology Co., Ltd. (Shanghai, China) Via NovaSeq Reagent Kits according to the manufacturer’s instructions (www.illumina.com (accessed on 10 December 2024)).

### 2.4. Sequence Quality Control and Genome Assembly

The data were analyzed on a free online platform, the Majorbio Cloud Platform. Briefly, the paired-end Illumina reads were trimmed of adaptors, and low-quality reads (length < 50 bp or with a quality value < 20 or having N bases) were removed by fastp (version 0.20.0. Metagenomic data were assembled using MEGAHIT (https://github.com/voutcn/megahit, version 1.1.2 (accessed on 10 December 2024)). Contigs with a length ≥ 300 bp were selected as the final assembly result, and the contigs were subsequently used for further gene prediction and annotation.

### 2.5. Gene Prediction, Taxonomic Analysis, Functional Annotation, and Statistical Analysis

Open reading frames (ORFs) from each assembled contig were predicted using Prodigal [17] and MetaGene [18]. A nonredundant gene catalog was constructed using CD-HIT [19] (version 4.6.1) with 90% sequence identity and 90% coverage. High-quality reads were aligned to nonredundant gene cataloges to calculate abundances of genes with 95% identity using SOAPaligner [20] (version 2.21). Representative sequences from the nonredundant gene catalog were aligned with the NR database with an e-value cutoff of 1 × 10^−5^ using Diamond (version 0.8.35) for taxonomic annotations. Cluster of orthologous groups of proteins (COG) annotations for the representative sequences were created Via Diamond against the eggNOG database. Kyoto encyclopedia of genes and genomes (KEGG) annotation was conducted using Diamond [21] against the kyoto encyclopedia of genes and genomes database. Carbohydrate-active enzyme annotation was conducted using hmmscan against the carbohydrate-active enzymes (CAZy) database, with an e-value cutoff of 1 × 10^−5^. Multiway ANOVA and one-way ANOVA were performed using SPSS (version 22.0, Chicago, IL, USA), and Duncan’s multiple range test was used for post hoc analysis of differences. QIIME software (version 1.9.1) was used to calculate the beta diversity distance matrix. Redundancy analysis (RDA) was conducted using the R language (version 4.5.1).

## 3. Results

### 3.1. Driving Factors of Soil Microbial Communities in the Rhizosphere of F. rubra

**Figure 1:**
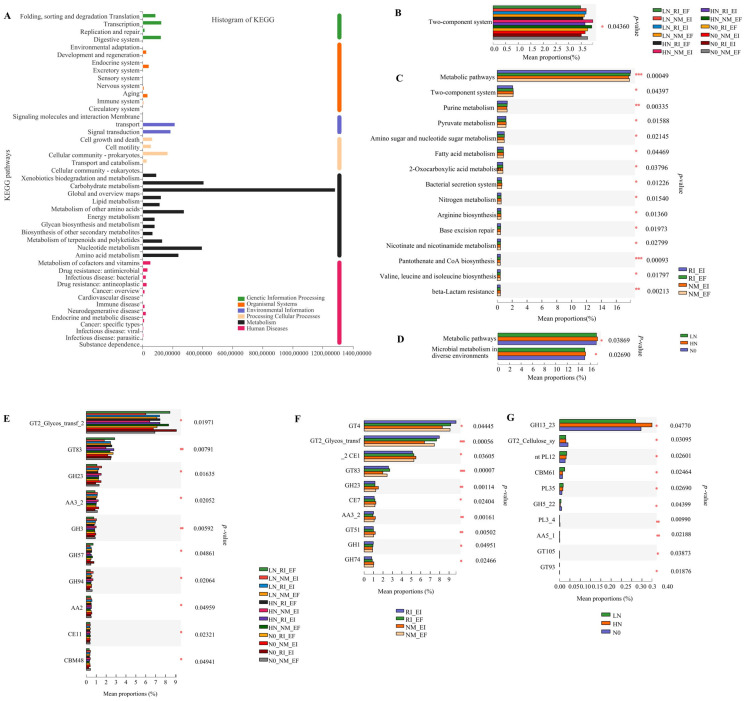
Functional annotation and difference analyses based on KEGG gene functional classification at pathway level 2 (**A**) and pathway level 3 (**B**–**D**) and CAZy (family) differences (**E**–**G**) in the rhizosphere soil microbial communities of *F. rubra*. Note: (**B**,**E**): nitrogen level and infection status; (**C**,**F**): infection status; (**D**,**G**): nitrogen level. Note: The red asterisk represents significant differences, * *p* < 0.05, ** *p* < 0.001, *** *p* < 0.0001.

The microbial community in the rhizosphere soil of *F. rubra* presented the greatest abundance of metabolic functional genes according to KEGG database analysis (Figure 1A). There were pronounced differences in CAZy function in the rhizosphere soil microbial communities of *F. rubra* with endophytic fungi infection and AMF colonization at the family level (*p* < 0.05, Figure 1F). There were few differences in the main functions of the microbial community in the rhizosphere soil associated with endophytic fungi and AMFs under different nitrogen levels at the category level and function level according to the EggNOG function database (Figure S1).

**Figure 2:**
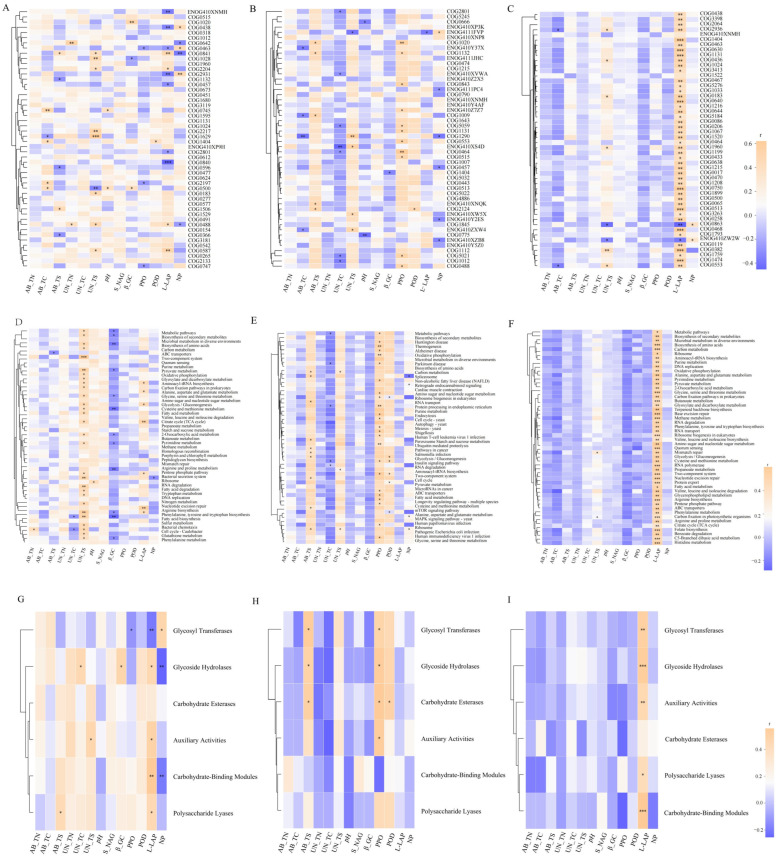
Correlations between soil microbial community functions and environmental factors in the rhizosphere of *F. rubra.* Note: (**A**–**C**): based on the COG functions of the community; (**D**–**F**): based on community KEGG pathway level 3 functions; (**G**–**I**): based on community CAZy (class level) functions; (**A**,**D**,**G**): bacteria; (**B**,**E**,**H**): fungi; and (**C**,**F**,**I**): archaea. Note: The asterisk represents significant differences, * *p* < 0.05, ** *p* < 0.001, *** *p* < 0.0001.

**Figure 3:**
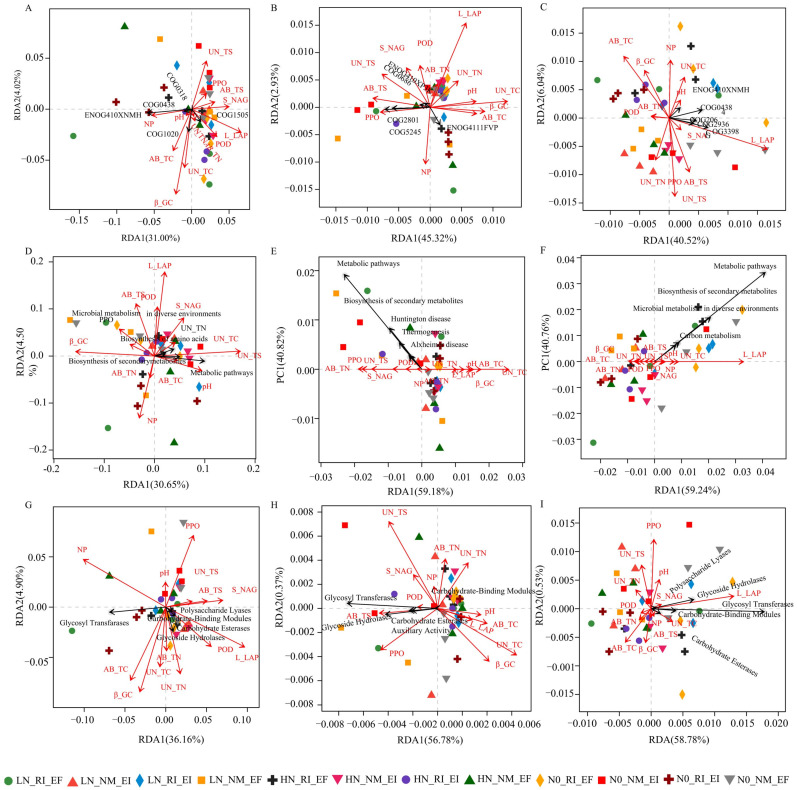
Functions of the rhizosphere soil microbial community and redundancy of environmental factors in *F. rubra.* Note: (**A**–**C**): eggNOG functional database COG functions; (**D**–**F**): KEGG pathway level 3 functions; (**G**–**I**): CAZy (class level) functions. (**A**,**D**,**G**): bacteria; (**B**,**E**,**F**): fungi; and (**C**,**F**,**I**): archaea.

The COG function and CAZy function of the rhizosphere soil bacterial, fungal and archaeal communities of *F. rubra* were correlated with environmental factors (*p* < 0.05, Figure 2). The function of CAZys (at the class level) in the archaeal community in the rhizosphere soil of *F. rubra* was positively correlated with soil L_LAP activity (*p* < 0.05, Figure 2I). The aboveground and belowground nutrient contents, soil pH and soil enzyme activities of *F. rubra* affected the functions of the soil bacterial, fungal and archaeal communities (Figure 3).

**Figure 4:**
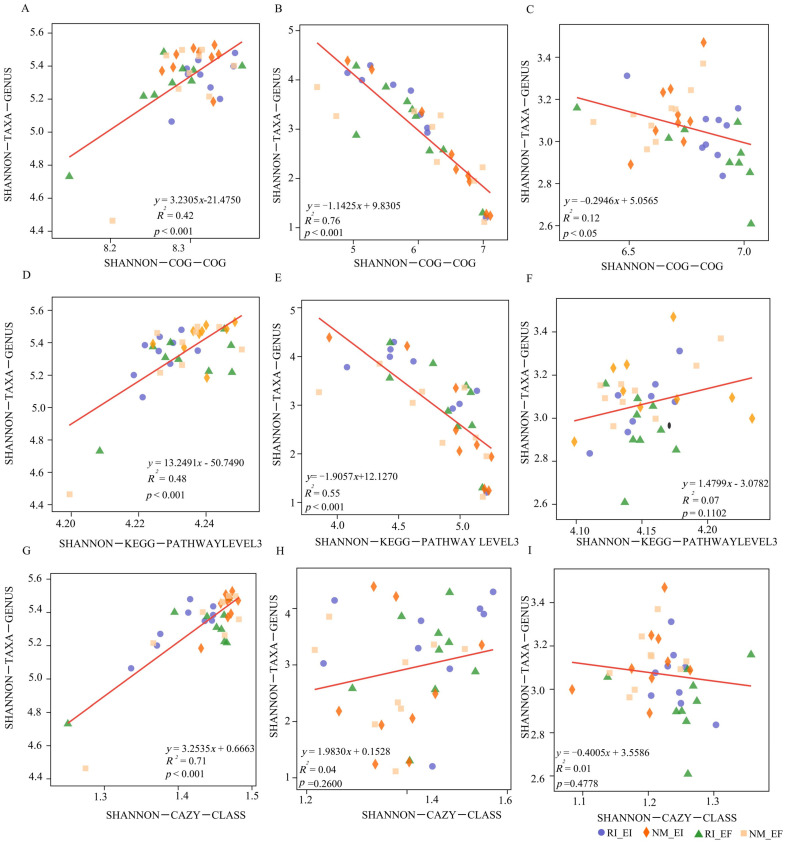
Regression analyses of rhizosphere soil community diversity and functional diversity under different infection statuses. Note: (**A**–**C**): eggNOG functional database; (**D**–**F**): KEGG functional database; (**G**–**I**): CAZy functional database. (**A**,**D**,**G**): bacteria; (**B**,**E**,**H**): fungi; and (**C**,**F**,**I**): archaea.

There were marked correlations between the community diversities of bacteria (*R*^2^ = 0.42, *p* < 0.01), fungi (*R*^2^ = 0.76, *p* < 0.01) and archaea (*R*^2^ = 0.12, *p* < 0.05) and their COG functional diversities (Figure 4). The diversity of the soil bacterial community was positively correlated with the functional diversities of the rhizosphere soil microbial community COG, KEGG (Pathway Level 3) and CAZy (class level) functions, the diversity of the soil fungal community was negatively correlated with the COG and KEGG (Pathway Level 3) functional diversities of the rhizosphere soil microbial community, and the diversity of the soil archaeal community was only negatively correlated with the COG functional diversities of the rhizosphere soil microbial community (Figure 4).

### 3.2. Functional Contributions and Metabolic Pathways of Different Groups of Soil Microbial Communities in the Rhizosphere of F. rubra

**Figure 5:**
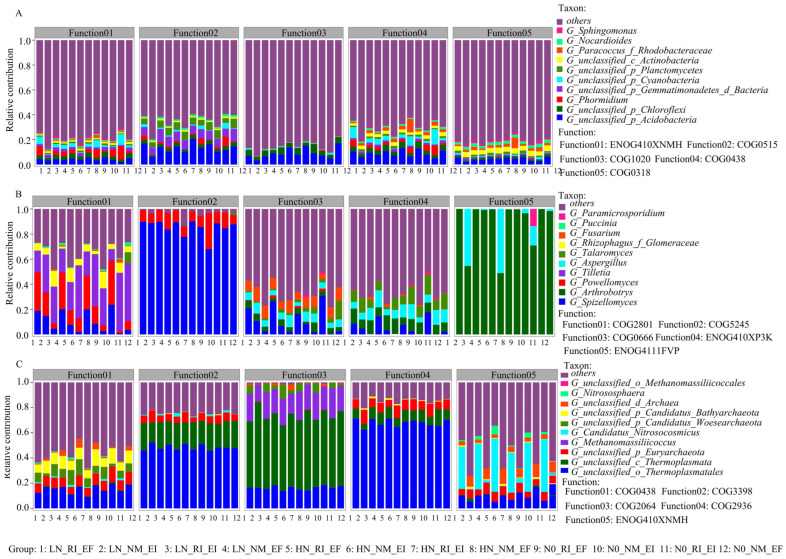
Contributions of COG functions to the bacterial (**A**), fungal (**B**) and archaeal (**C**) communities in the rhizosphere soil of *F. rubra* infected with endophytic fungi and AMFs at different nitrogen levels. Note: COG0438: glycosyltransferase; COG0318: AMP-dependent synthase and ligase; COG5245: heavy chain; COG0666: ankyrin repeat sequence; ENOG410XP3K: positively regulates the activity of the negative end-oriented microtubule motility protein, and dynamic protein-mediated microtubule sliding can be enhanced by targeting the positive end of the microtubule; ENOG4111FVP, chelate protein; COG1020, nonribosomal peptide synthetase; and GOG0515: serine threonine protein kinase.

**Figure 6:**
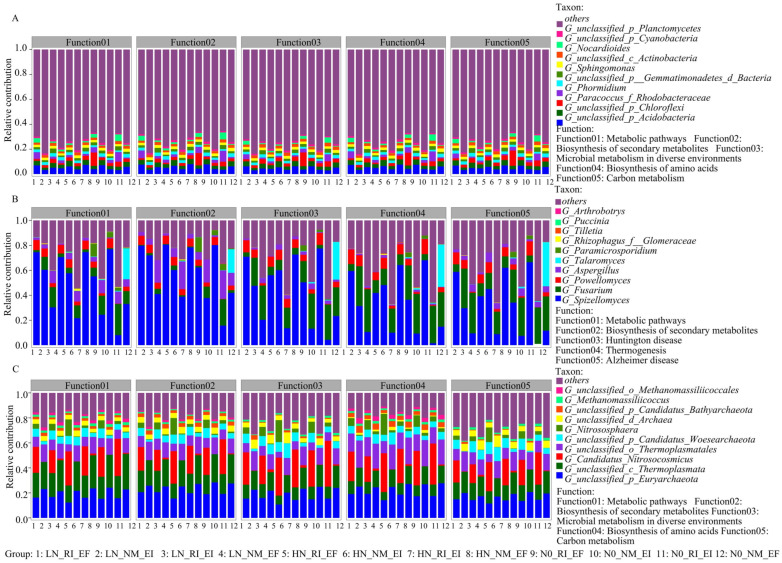
Contributions of KEGG pathway level 3 functions to the bacterial (**A**), fungal (**B**) and archaeal (**C**) communities in the rhizosphere soil of *F. rubra* infected with endophytic fungi and AMFs at different nitrogen levels.

**Figure 7:**
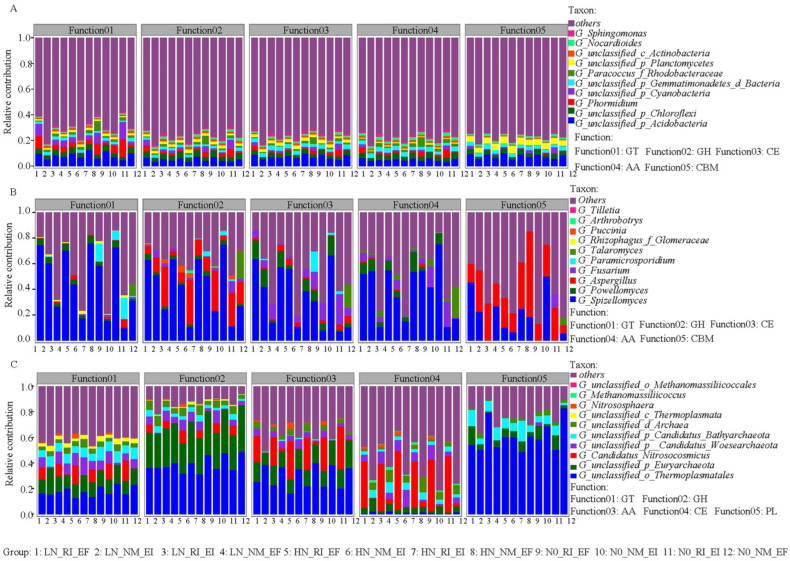
Contributions of CAZy (class) functions to the bacterial (**A**), fungal (**B**) and archaeal (**C**) communities in the rhizosphere soil of *F. rubra* infected with endophytic fungi and AMFs at different nitrogen levels. Note: GT: glycosyl transferase; GH: glycoside hydrolase; CE: carbohydrate esterase; AA: auxiliary activity; CBM: carbohydrate-binding module; and PL: polysaccharide lyase.

Proteobacteria was the largest contributor to COG function, KEGG pathway level 3 function, and CAZy (class level) function (Figure 5, Figure 6 and Figure 7). In the fungal community, Chytridiomycota and Basidiomycota contributed mainly to the COG2801 (retrotransposon protein) function, KEGG pathway level 3 function, and CAZy (class level) function. In the archaeal community, Euryarchaeota was the main contributor to COG function and KEGG pathway level 3 function (Figure 5, Figure 6 and Figure 7). The contribution of *Aspergillus* fungi to ENOG4111FVP function (33.33%) was greater than that of the other treatments (Figure S2). Moreover, the contributions of AMF colonization to the KEGG (pathway level 3) functions of the bacterial and archaeal communities in the rhizosphere soil of *F. rubra* were greater than those in the rhizosphere soil of *F. rubra* infected with endophytic fungi (Figure S3). The CAZy functions of the bacterial and archaeal communities in the rhizosphere soil of *F. rubra* at high nitrogen levels were stronger than those in the rhizosphere soil microorganisms of *F. rubra* at low nitrogen levels (Figure S4).

### 3.3. The Metabolism Pathways of Soil Microbial Communities in the Rhizosphere of F. rubra

**Figure 8:**
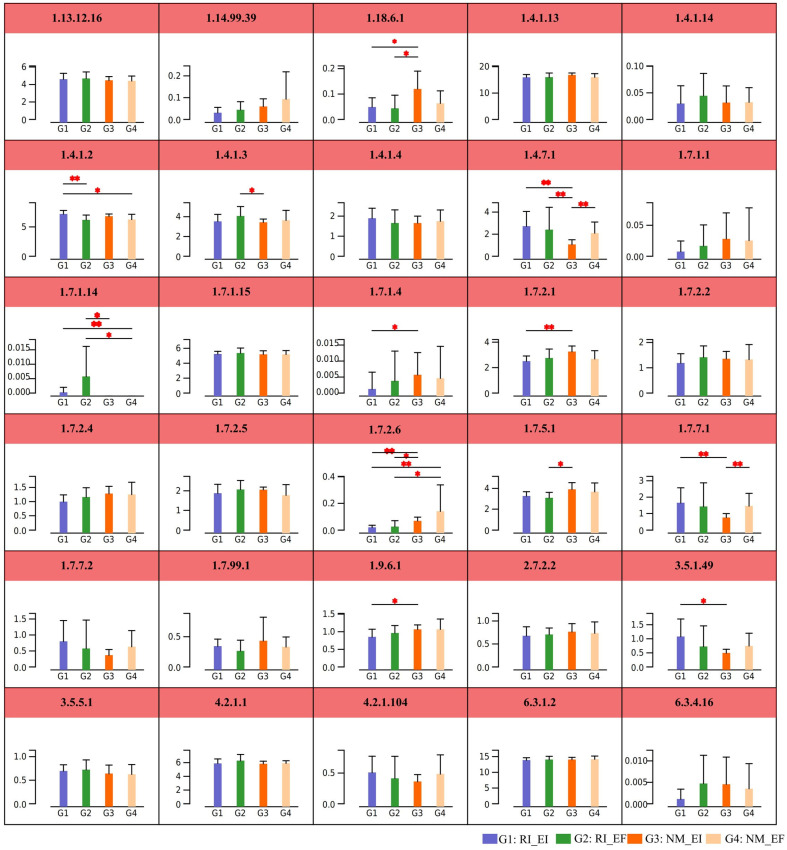
Differences of nitrogen metabolism in rhizosphere soil microbial communities of *F. rubra* under different fungal infection treatments. Note: G1represent included RI colonization (RI) and endophytic fungi infected (EI); G2 represent included RI colonization (RI) and endophytic fungi free (EF), G3 represent no RI colonization (NM) and endophytic fungi infected (EI); G4 represent no RI colonization (NM) and endophytic fungi free (EF). Note: The red asterisk represents significant differences, * *p* < 0.05, ** *p* < 0.001.

Endophytic fungal and AMF colonization had marked effects on the nitrogen metabolism pathways of the rhizosphere soil microbial community of *F. rubra* (Figure 8 and Figure S5). Among them, the relative abundances of nitrogenase and iron oxidoreductase (ATP hydrolysis) in rhizosphere soil microorganisms under endophytic NM_EI fungal colonization were greater than those under AMF colonization (RI_EF) (*p* < 0.05). The relative abundances of glutamate dehydrogenase and glutamate synthase (ferredoxin) in RI_EF treatment were greater than those in the NM_EI treatment (*p* < 0.05) (Figure 8).

## 4. Discussion

### 4.1. Functions and Factors Influencing the Soil Microbial Communities in the Rhizosphere of F. rubra

In this study, the abundance of metabolic functional genes in the rhizosphere soil microbial community was the highest, followed by that of genes involved in carbohydrate metabolism, and the lowest abundance was observed for genes involved in the metabolism of terpenes and polyketones, according to the analysis of the KEGG database. These results indicated that dual colonization with endophytic fungi and AMFs at different nitrogen levels first affected the production of secondary metabolites in the rhizosphere microbial community of *F. rubra* and then affected carbohydrate metabolism [10]. The CAZy functions of the endophytic fungus-infected and AMF-infected *F. rubra* rhizosphere soil microbial communities at the class level were consistent with the results of related research [22], suggesting that glycoside hydrolases and carbohydrate esterases functioned at this level. There was a positive correlation between the KEGG functions of the rhizosphere soil bacterial community and the belowground sulfur content of *F. rubra*. The relationships between the KEGG functional profiles of soil bacterial communities and the sulfur content are influenced by multiple factors, including sulfur-oxidizing bacterial diversity and abundance, soil depth, and microbial community enzyme activity [23,24,25]. A previous study revealed that exogenous sulfur addition could enhance the glutathione reductase pathway, which could explain the marked positive correlation with KEGG function [26]. Furthermore, the glycosyltransferase function of the rhizosphere soil bacterial community was positively correlated with both soil NP activity and the glycoside hydrolase. Studies have shown that the presence of phosphoryl regulators in some bacteria could affect the expression of phosphatases.

### 4.2. Relationships Between Microbial Community Diversity and Function in the Rhizosphere Soil of F. rubra

The diversity of soil microbial communities is closely related to their functions [27]. In this study, the diversities of bacteria (*R*^2^ = 0.42, *p* < 0.001), fungi (*R*^2^ = 0.76, *p* < 0.001) and archaea (*R*^2^ = 0.12, *p* < 0.05) in the rhizosphere soil of *F. rubra* were correlated with their COG functional diversities. Among them, the COG functions increased with increasing bacterial community diversity, whereas the correlation between fungal and archaeal community diversity and COG function was the opposite. This result could have arisen because bacteria of different genera have different functions [28]. Fungi are mainly responsible for the decomposition of carbon-containing organic matter [29,30,31]. In contrast, bacteria mainly use nitrogenous organic matter [32]. Soil microorganisms are responsible for maintaining soil viability, and play important roles in maintaining the overall service functions of soil ecosystems [27]. When the ecological environment of soil microorganisms is disturbed, the number, activity, diversity and community structure of microorganisms are affected [22,33,34]. In addition, the abundances of soil bacteria in the rhizosphere soil of *F. rubra* were greater than those in the control, indicating that the AMF-infected rhizosphere soil of *F. rubra* was conducive to the growth and reproduction of microorganisms in this study. Changes in microbial communities could indirectly affect the diversity of their metabolic functions. According to KEGG database analysis, AMF-infected *F. rubra* rhizosphere soil treatment altered some metabolic functions of the soil microorganisms. Similarly, a study revealed that there was a marked positive correlation between soil bacterial community functional characteristics and community diversity [35]. Therefore, the functional diversity of bacteria, fungi and archaea in the rhizosphere soil of *F. rubra* was closely related to the species composition.

### 4.3. Effects of Endophytic Fungi and AMF Colonization on the Metabolic Pathways of the F. rubra Rhizosphere Soil Microbial Community

Soil microorganisms drive a range of interrelated carbon and nitrogen conversion processes, including carbon and nitrogen degradation and fixation, methane metabolism, nitrification and denitrification, which are essential for maintaining soil quality and crop growth. Studies have shown that AMFs can affect bacterial communities In Vitro culture conditions [34,35,36]. In consistant, we found AMF colonization enriched the metabolic pathways of the bacterial and archaeal communities in the rhizosphere soil. Under different nitrogen levels, the relative abundances of enzymes involved in nitrogen metabolism pathways differed among the *F. rubra* rhizosphere soil microbial communities, specifically those infected by endophytic fungi and those colonized by AMFs. In this study, glutamate synthase was the most abundant nitrogen metabolism pathway in the rhizosphere soil microbial community of *F. rubra*. The microbial community of *F. rubra* rhizosphere soil infected with endophytic fungi and AMFs presented increased abundances of genes encoding enzymes involved in nitrogen metabolism pathways, such as glutamate synthase and dinitrogen oxidoreductase, indicating that these enzymes have the potential to improve the nitrogen fixation capacity of soil microorganisms. The microbial community in the rhizosphere soil associated with endophytic fungi and AMF colonization increased nitrogen metabolism in the rhizosphere soil, indicating that the microbes involved in nitrogen metabolism and nitrogen fixation in the rhizosphere soil changed after the input of different forms of nitrogen. Rhizosphere soil harbored a unique nitrifying bacterial community and abundant functional genes related to nitrogen cycling, which could provide abundant microbial resources for soil nitrogen fixation [37]. These findings will contribute to a deeper understanding of the mechanisms by which plant-symbiotic fungi influence the metabolic pathways of microbial communities in the host rhizosphere soil.

## 5. Conclusions

AMF colonization enhanced the metabolic pathways, secondary metabolite biosynthesis, microbial metabolism, amino acid biosynthesis, and carbon metabolism processes in the rhizosphere soil bacterial and archaeal communities. The effects of endophytic fungi and AMFs on the functions and metabolic pathways of the symbiotic rhizosphere soil microbial communities were heterogeneous. This study provides important scientific evidence for a deeper understanding of how plant-fungal symbiotic relationships contribute to the maintenance of soil ecosystem functions.

## Data Availability

The raw data supporting the conclusions of this article will be made available by the authors on request.

## Supplementary Materials

The following supporting information can be downloaded at: https://www.mdpi.com/article/10.3390/microorganisms13122735/s1, Figure S1: Functional composition analysis of COG and CAZy at category and function levels in rhizosphere soil microbial community in *F. rubra*; Figure S2: Functional contributions of bacterial (A,D) fungal (B,E) and archaea (C,F) communities to COG in rhizosphere soil of *F. rubra*; Figure S3: Functional contributions of bacterial (A,D) fungal (B,E) and archaea (C,F) communities to KEGG Pathway Level 3 in rhizosphere soil of *F. rubra*; Figure S4: Functional contributions of bacterial (A,D) fungal (B,E) and archaea (C,F) communities to CAZy (class) in rhizosphere soil of *F. rubra*; Figure S5: Nitrogen metabolism pathway of the rhizosphere soil microbial community infected by endophytic fungi and AMF in *F. rubra* under different nitrogen levels based on KEGG database.
