# Supplementary material for: Effects of Endophytic Fungi and Arbuscular Mycorrhizal Fungi on Microbial Community Function and Metabolic Pathways in the Rhizosphere Soil of Festuca rubra
Source: Microorganisms. 2025 Nov 30;13(12):2735. doi: 10.3390/microorganisms13122735 (PMC12735325; doi:10.3390/microorganisms13122735)
Supplement: Supplementary file 1 [file microorganisms-13-02735-s001.zip › microorganisms-3987521 supplementary_conversion.pdf]

Supporting Information for

Effects of endophytic fungi and arbuscular mycorrhizal fungi on microbial community function and metabolic pathways in the rhizosphere soil of *Festuca rubra*

Zhengming Luo[1,2], Yanying Zhou[1], Xuerong Wang[3], Lei He[4] and Tong Jia[3*]

[1] Department of Geography, Xinzhou Normal University, Xinzhou 034000, China

[2] Soil Health Laboratory of Shanxi Province, Shanxi Agricultural University, Taiyuan 030031, China

[3] Shanxi Key Laboratory for Ecological Restoration of Loess Plateau, Institute of Loess Plateau, Shanxi University, Taiyuan 030006, China

[4] Shanxi Forestry and Grassland General Engineering Station, Taiyuan 030021, China

* **Correspondence: jiatong@sxu.edu.cn**

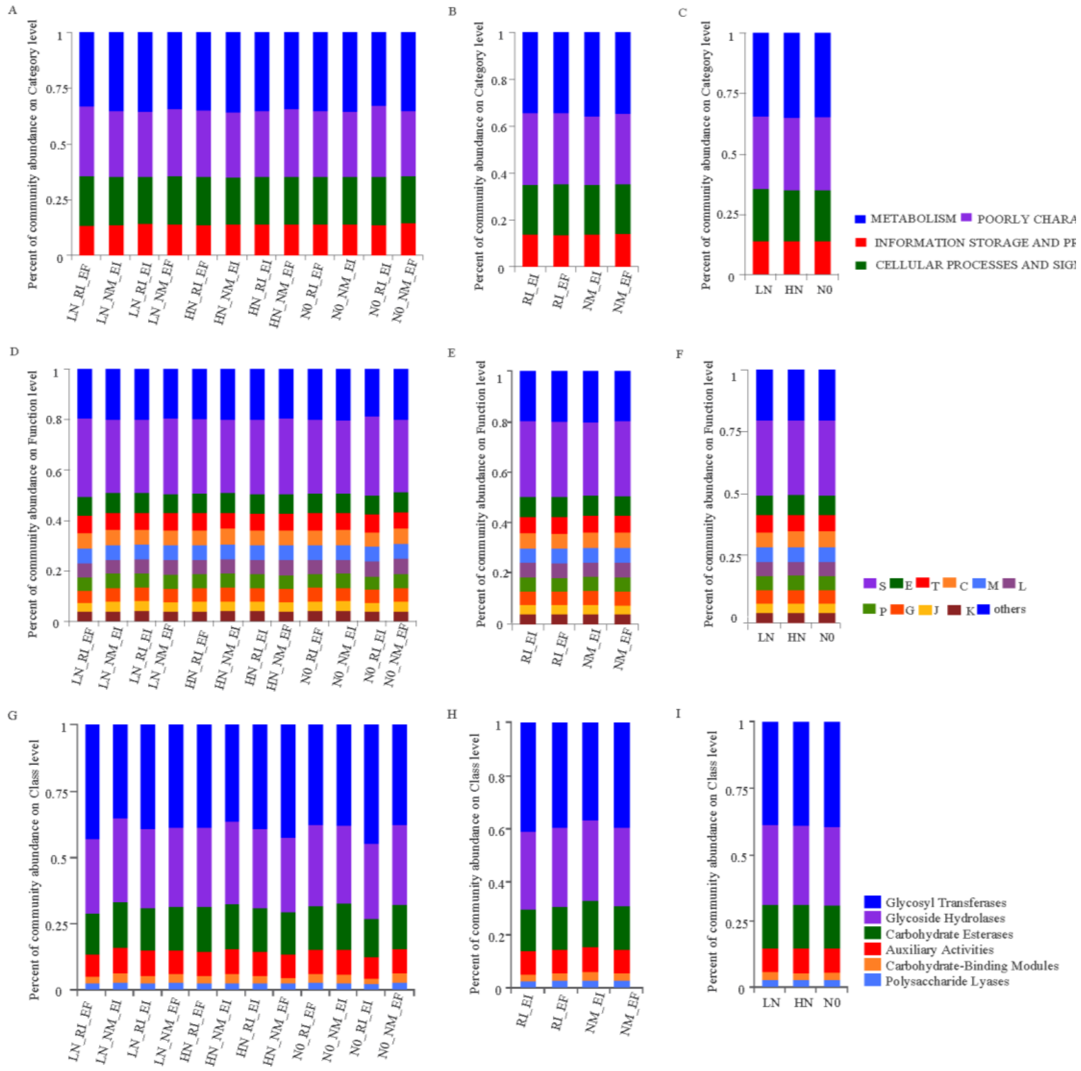

Fig. S1 Functional composition analysis of COG and CAZy at category and function levels in rhizosphere soil microbial community in *F. rubra*.

Note: E, amino acid transport and metabolism; T, signal transduction mechanisms; C, energy production and conversion; M, cell wall/membrane/envelope biogenesis; L, replication, recombination and repair; P, inorganic ion transport and metabolism; G, carbohydrate transport and metabolism; J, translation, ribosomal structure and biogenesis; K, transcription; S, function unknown

Fig. S2

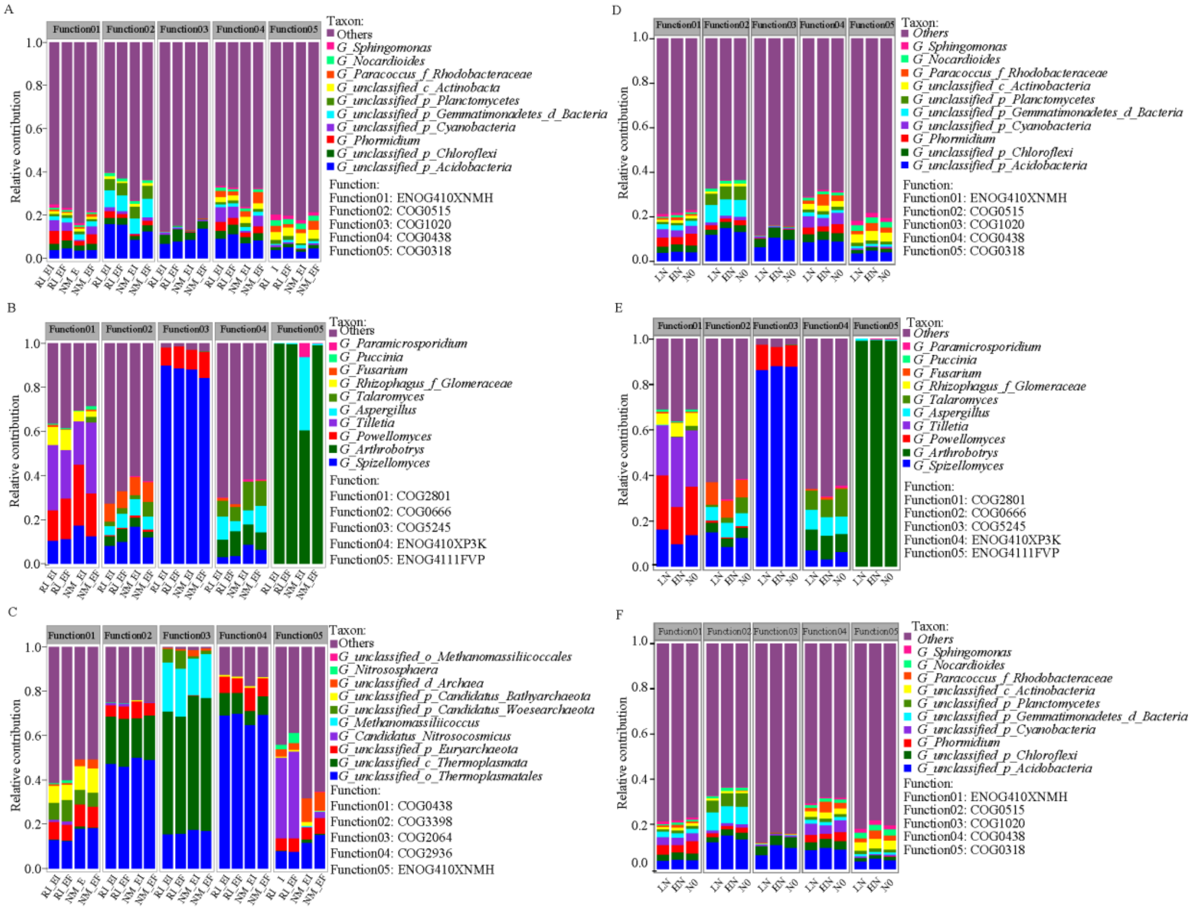

Fig. S2 Functional contributions of bacterial (A, D) fungal (B, E) and archaea (C, F) communities to COG in rhizosphere soil of *F. rubra*

Fig. S3

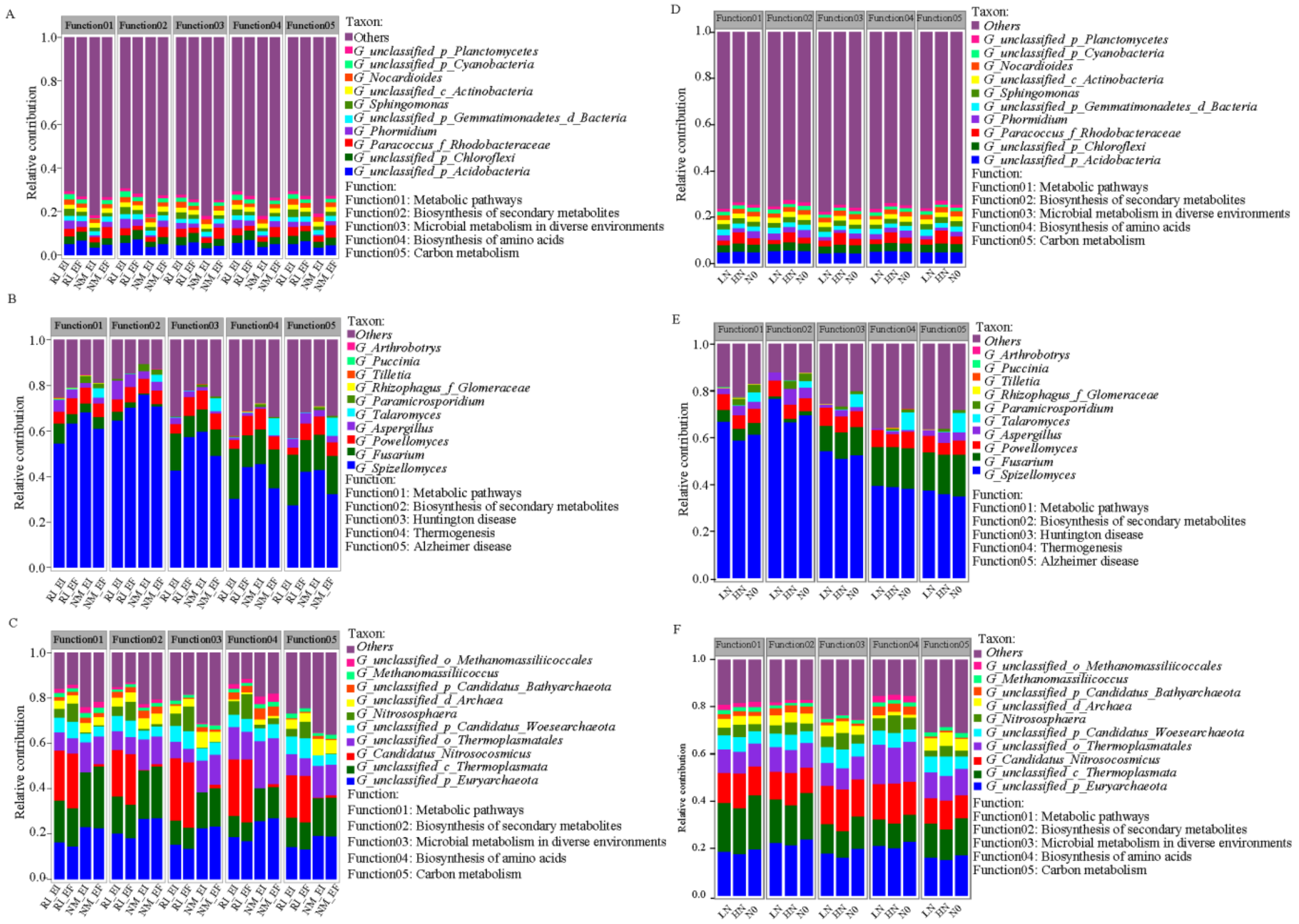

Fig. S3 Functional contributions of bacterial (A, D) fungal (B, E) and archaea (C, F) communities to KEGG Pathway Level 3 in rhizosphere soil of *F. rubra*

Fig. S4

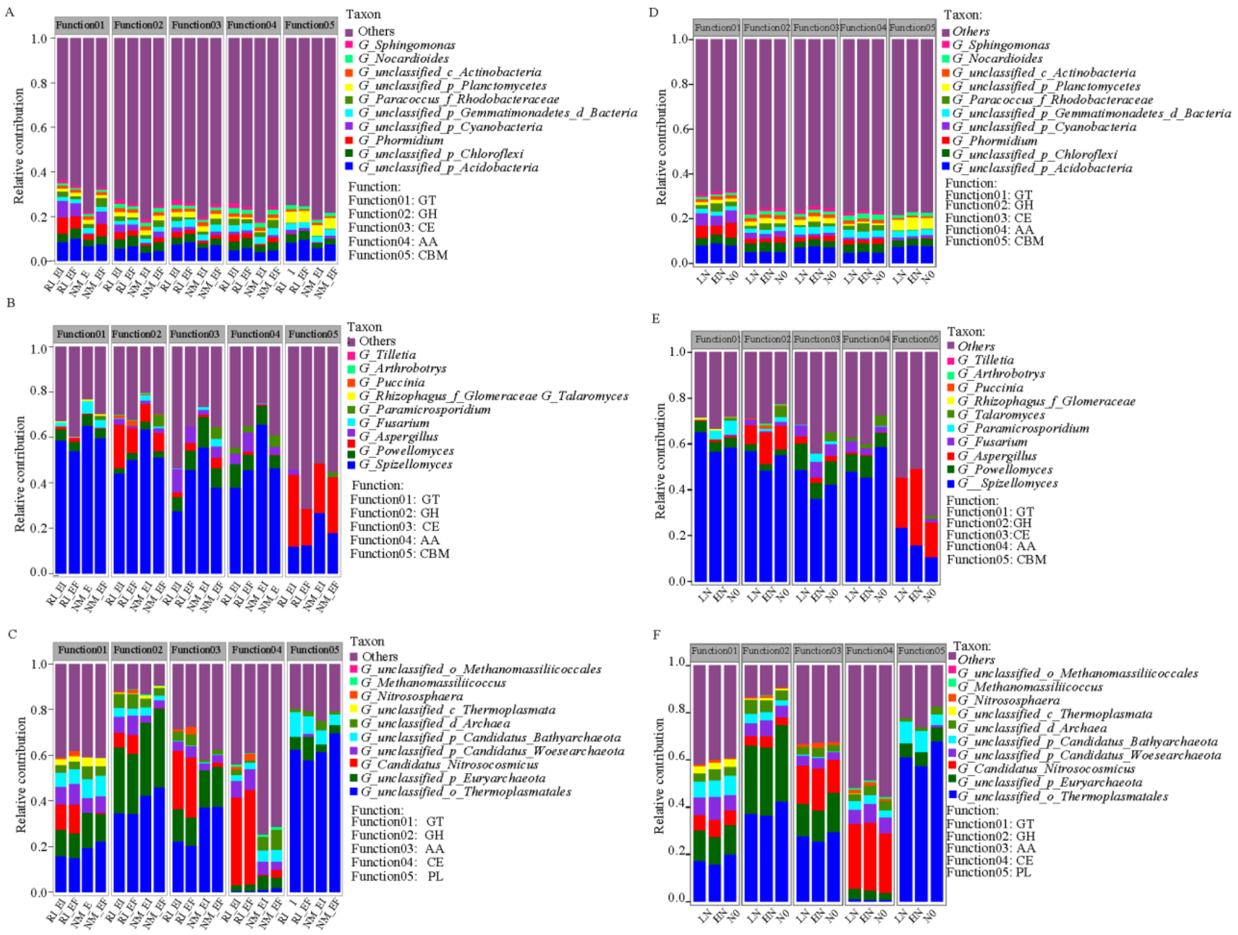

Fig.S4 Functional contributions of bacterial (A, D) fungal (B, E) and archaea (C, F) communities to CAZy (class) in rhizosphere soil of *F. rubra*

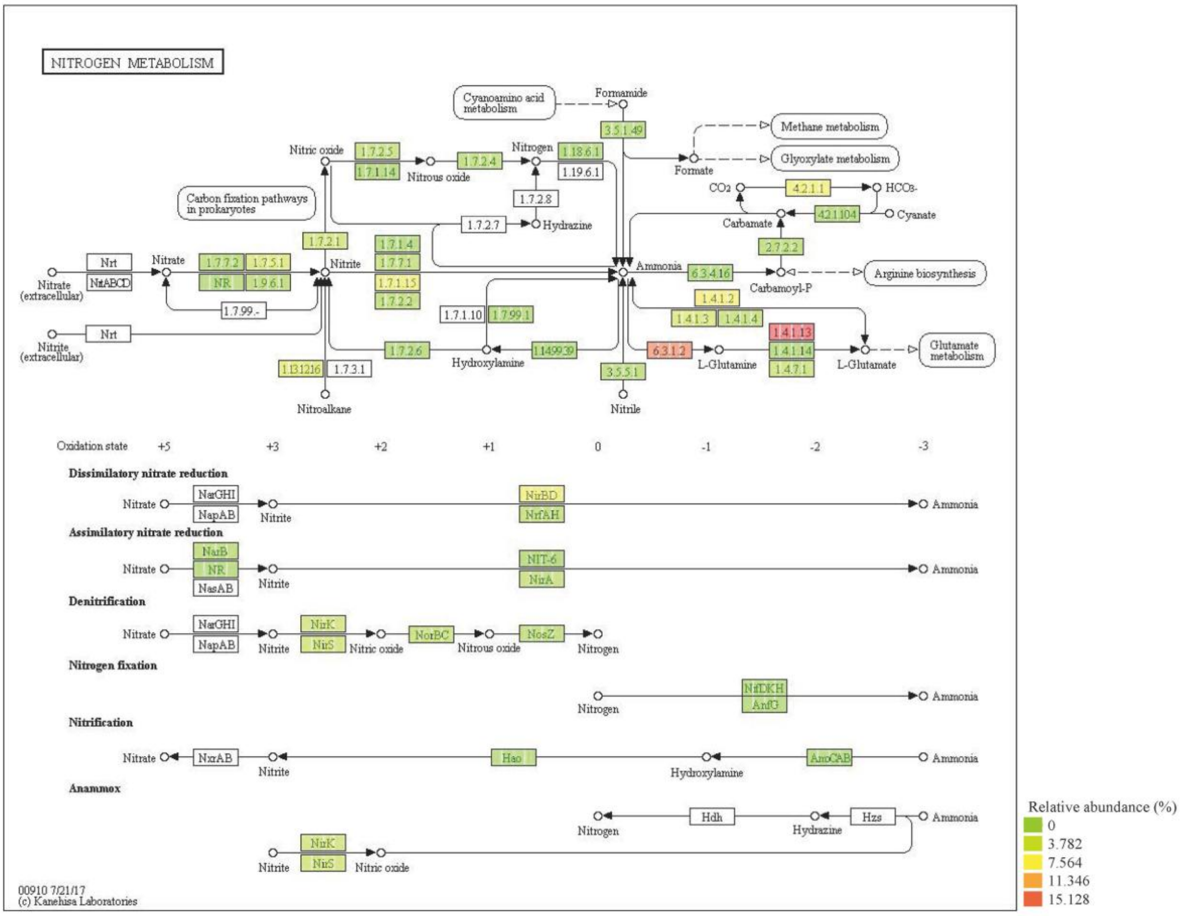

Fig. S5 Nitrogen metabolism pathway of the rhizosphere soil microbial community infected by endophytic fungi and AMF in *F. rubra* under different nitrogen levels based on KEGG database

Note: The boxes with filled colors in the figure represent a group of samples, and the depth of the color represents the variation in enzyme abundance in different groups. The change in color from green to red represents the relative abundance.The 17 KEGG pathways contained: map00220, arginine biosynthesis; map00230, purine metabolism; map00250, alanine, aspartate and glutamate metabolism; map00260, glycine, serine and threonine metabolism; map00460, cyanoamino acid metabolism; map00630, glyoxylate and dicarboxylate metabolism; map00910，nitrogen metabolism; map01100， metabolic pathways; map01110， biosynthesis of secondary metabolites; map01120, microbial metabolism in diverse environments; map01200, carbon metabolism; map01230, biosynthesis of amino acids; map04964, proximal tubule bicarbonate reclamation; map04966, collecting duct acid secretion; map04974, protein digestion and absorption; map05110, vibrio cholerae infection; map05120，epithelial cell signaling in helicobacter pylori infection）。Seven

modules were: M00029, urea cycle; M00175, nitrogen fixation, nitrogen ammonia; M00528, nitrification; M00530, dissimilatory nitrate reduction; M00531, assimilatory nitrate reduction; M00532, photorespiration; M00804, complete nitrification.
